# Regulation of Chromatin Structure During Neural Development

**DOI:** 10.3389/fnins.2018.00874

**Published:** 2018-12-11

**Authors:** Yusuke Kishi, Yukiko Gotoh

**Affiliations:** ^1^Graduate School of Pharmaceutical Sciences, The University of Tokyo, Tokyo, Japan; ^2^International Research Center for Neurointelligence (WPI-IRCN), The University of Tokyo, Tokyo, Japan

**Keywords:** neural development, chromatin, chromatin conformation capture (3C), topologically associated domain (TAD), A/B compartments

## Abstract

The regulation of genome architecture is a key determinant of gene transcription patterns and neural development. Advances in methodologies based on chromatin conformation capture (3C) have shed light on the genome-wide organization of chromatin in developmental processes. Here, we review recent discoveries regarding the regulation of three-dimensional (3D) chromatin conformation, including promoter–enhancer looping, and the dynamics of large chromatin domains such as topologically associated domains (TADs) and A/B compartments. We conclude with perspectives on how these conformational changes govern neural development and may go awry in disease states.

## Introduction

The human and mouse genomes consist of ∼6 and ∼5 billion base pairs, respectively, and are packaged in chromosomes that are contained within a nucleus with a diameter of only ∼5 μm. Chromosomes possess multilayered structures that can be broadly classified on the basis of classical cytological and biochemical analyses either as euchromatin, an open chromatin state characteristic of gene-rich regions, or as heterochromatin, a closed chromatin state characteristic of gene-poor regions. At a higher level of resolution, local associations between gene promoters and other regulatory elements, such as enhancers, define the structural relations within active transcriptional domains (Vernimmen and Bickmore, 2015).

**FIGURE 1:**
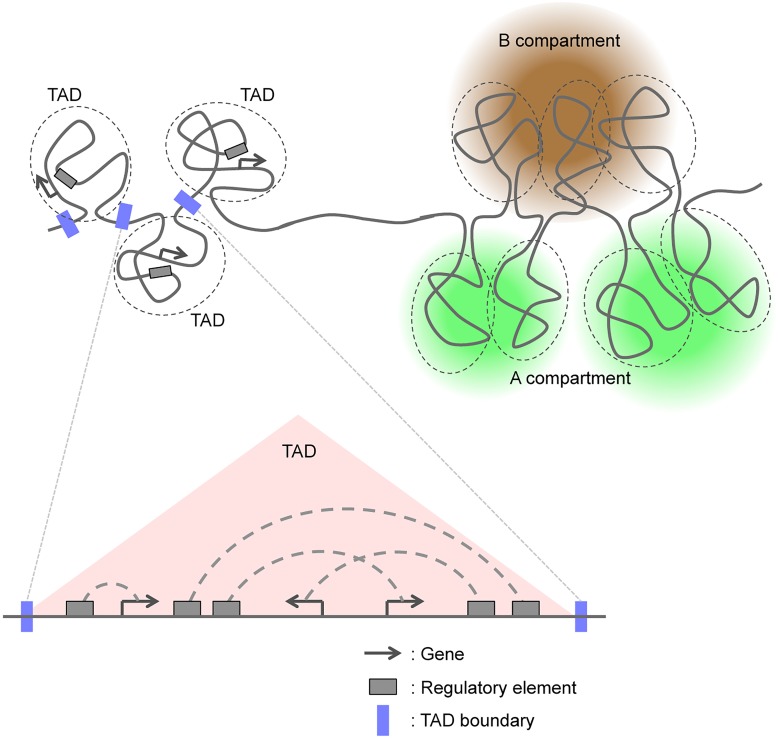
Three-dimensional genome organization based on TADs and A/B compartments.

High-throughput chromatin conformation capture (3C) techniques have recently allowed the categorization of chromosomal domains into two major classes (Figure 1; van de Werken et al., 2012; Bonev and Cavalli, 2016; Dekker and Mirny, 2016; Dixon et al., 2016; Hansen et al., 2018). In this review, we first briefly summarize advances in our understanding of the molecular mechanisms that regulate the formation of TADs and A/B compartments. We then address recent studies that have examined changes in genomic interactions and three-dimensional (3D) genome organization including TADs and A/B compartments during mammalian neural development, and we discuss how these chromosomal changes regulate this process.

## Formation of Tads and A/B Compartments

Recent studies have revealed some molecular mechanisms underlying the formation of TADs. The zinc-finger DNA-binding protein CTCF and the ring-shaped cohesin complex bind to many boundaries between TADs (Dixon et al., 2012; Nora et al., 2012; Rao et al., 2014), and some studies have proposed that “loop extrusion” mediated by the cohesin complex and the convergent orientation of CTCF binding play a role in TAD formation (Sanborn et al., 2015; Fudenberg et al., 2016). Real-time imaging revealed that the condensin complex, which belongs to the same Smc family as the cohesin complex, indeed induced DNA loop extrusion *in vitro* (Ganji et al., 2018). Importantly, forced degradation of CTCF or Rad21, an essential component of the cohesin complex, with the use of the auxin-induced rapid degradation system, resulted in the almost complete elimination of TADs (Nora et al., 2017; Rao et al., 2017). Conditional knockout of the cohesin-loading factor Nipbl or Scc4 also induced deformation of TADs (Haarhuis et al., 2017; Schwarzer et al., 2017). These observations have suggested that CTCF and the cohesin complex are essential for the establishment of TADs. However, even though TADs were essentially eliminated in cells depleted of CTCF or Rad21, A/B compartments were largely unaffected (Nora et al., 2017; Rao et al., 2017). This finding indicates that A/B compartmentalization of mammalian chromosomes emerges independently of proper insulation of TADs, even though TADs serve as units of A/B compartments. Interestingly, acute loss of cohesin had only limited effects on gene expression and the distribution of various histone modifications (Rao et al., 2017; Schwarzer et al., 2017), which may suggest that regulatory interactions are somewhat preserved after the loss of TADs.

With regard to A/B compartments, heterochromatin has been proposed to serve as a driver of compartmentalization. Lamina-associated domains (LADs), defined as genomic regions that contact the nuclear lamina, constitute heterochromatin at the nuclear periphery (van Steensel and Belmont, 2017). LADs revealed by a method known as DamID (DNA adenine methyltransferase identification) analysis showed cell-to-cell heterogeneity and a strong correlation with the B compartment (Rao et al., 2014; Kind et al., 2015). Given that the nuclear lamina provides a platform for chromatin reassembly during the M-to-G_1_ phase transition of the cell cycle (Güttinger et al., 2009), LAD formation may underlie compartmentalization of heterochromatin domains and the B compartment, although this is still under debate (Falk et al., 2018). Another emerging feature of heterochromatin domains is phase separation into liquid droplets mediated by heterochromatin protein 1 (HP1) (Larson et al., 2017; Strom et al., 2017). Liquid phase separation is thought to provide a basis for the formation of membrane-less structures (Boeynaems et al., 2018). The B compartment can be considered as such a membrane-less structure given the enrichment of histone H3 methylated at lysine-9 (H3K9) in this compartment (Rao et al., 2014), which provides a platform for HP1 binding and oligomerization required for liquid phase separation, as supported by a recent modeling experiment (Falk et al., 2018).

Although these various studies have elucidated the framework for 3D organization of the genome, many questions regarding TAD formation – including the role of transcription, whether loop extrusion is asymmetric, and the relevance of DNA replication – remain unanswered. In addition, the mechanisms underlying A/B compartmentalization remain largely elusive. A key unanswered question regarding genome architecture is, how do local and global-scale associations, including those mediated by A/B compartments and TADs, govern changes in transcription and cell fate during development. In this review, we focus on studies on neural development in an attempt to tackle this question.

## Global Changes in 3D Genome Organization During Neural Differentiation

### Global Compaction During Neural Differentiation

The 3D architecture of chromatin changes markedly during the neural development of pluripotent stem cells. Assays based on micrococcal nuclease (MNase) or DNase I accessibility or on histone extraction have revealed that the chromatin state is globally open in embryonic stem cells (ESCs) and becomes condensed during differentiation into neural progenitor cells (NPCs) (Meshorer et al., 2006). Even among NPCs, the loss of neurogenic potential during neocortical development is associated with chromatin condensation on a large scale (Kishi et al., 2012a; Tyssowski et al., 2014). The “openness” of chromatin may be related to differentiation potential (“stemness”) in these cells, given that the factors responsible for global chromatin accessibility – Chd1 in ESCs and Hmga in NPCs – are also required for differentiation potential (Gaspar-Maia et al., 2009; Kishi et al., 2012a). Chromatin state also undergoes pronounced changes during neuronal differentiation of NPCs. For example, the number and shape of chromocenters – heterochromatin foci strongly stained with DNA-intercalating dyes – change during neuronal differentiation (Billia et al., 1992; Solovei et al., 2004, 2009; Clowney et al., 2012; Le Gros et al., 2016). Likewise, an increase in the deposition of the active histone mark H3K4me3 (trimethylated lysine-4 of histone H3) at chromocenters, accompanied by an increase in transcription of major satellites, is also observed during neuronal differentiation in the neocortex (Kishi et al., 2012b). Recent examinations of chromatin accessibility by the assay for transposase-accessible chromatin with high-throughput sequencing (ATAC-seq), DNase-seq, and formaldehyde-assisted isolation of regulatory elements (FAIRE)-seq have revealed progressive changes in chromatin openness during neuronal differentiation processes (Frank et al., 2015; Thakurela et al., 2015; de la Torre-Ubieta et al., 2018; Preissl et al., 2018), which would link chromatin accessibility to the genome architecture associated with these processes.

### Loss of Active-Domain and Increase in Inactive-Domain Interactions During Neural Differentiation

**FIGURE 2:**
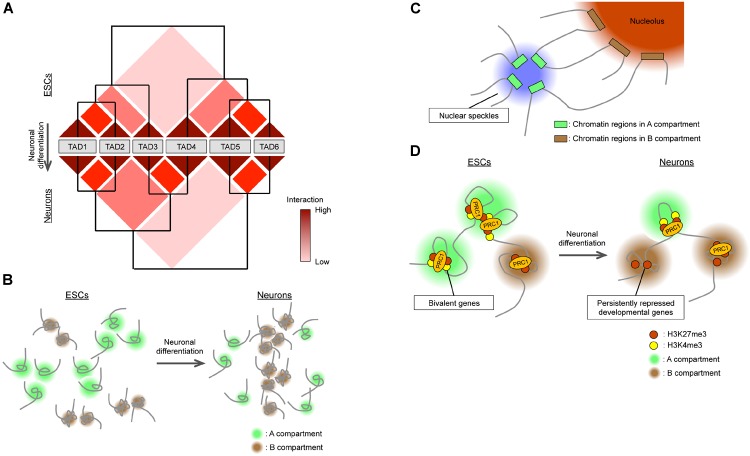
Global changes in 3D genome organization during neural differentiation. **(A)** TADs are organized in a hierarchical manner, and their reorganization accompanies neural differentiation. **(B)** During neural differentiation from ESCs, interactions within the A compartment decrease while those within the B compartment increase. The size of the A compartment also decreases during neural differentiation. **(C)** Nuclear speckles and nucleoli act as hubs for interactions within A and B compartments, respectively. **(D)** In ESCs, bivalent genes interact with each other *via* PRC1 in the A compartment. Neural differentiation is accompanied by the loss of PRC1-mediated interactions between bivalent genes, with the genes becoming persistently repressed and relocating to the B compartment.

So, how are TADs and A/B compartments regulated during neural development? TADs are structurally dynamic overall (Hansen et al., 2018), but TAD boundaries, on the other hand, are stable for many cell divisions and invariant across diverse cell types or lineages (Nora et al., 2012; Rao et al., 2014; Dixon et al., 2015, 2016). Indeed, differentiation of ESCs into NPCs and then into neurons is not accompanied by changes in the boundaries of most TADs (Fraser et al., 2015). Rather, inter-TAD interactions as well as chromatin interactions within TADs (sub-TAD or intra-TAD level, including chromatin looping) change during differentiation (Fraser et al., 2015; Dixon et al., 2016, 2015). Fraser et al. (2015) proposed that TADs are organized into meta-TADs in a hierarchical manner, and that neural differentiation of ESCs is accompanied by the rearrangement of meta-TAD components (Figure 2A). A fraction of inter-TAD rearrangement is associated with changes in gene expression within TADs (Fraser et al., 2015), and TAD allocation to A/B compartments changes during differentiation (Dixon et al., 2015).

In contrast to TADs, A/B compartments are differentially regulated during neural development. Recent studies have examined and compared genome-wide 3D chromatin organization during neural differentiation from ESCs (Dixon et al., 2015; Bonev et al., 2017) by Hi-C analysis, which allows the detection of complete “all versus all” long-distance chromatin interactions across the entire genome (Lieberman-Aiden et al., 2009). One study (Dixon et al., 2015) found that the total size of the A compartment in differentiated cells including NPCs was reduced by 5% compared with that in ESCs (Figure 2B). This finding appears to be consistent with the global condensation of the chromatin state observed when ESCs differentiate into neural cells mentioned above. Another study (Bonev et al., 2017) based on higher-resolution Hi-C analysis (maximum of 750 bp) found that interactions within the A compartment decreased during the ESC-to-NPC transition, interactions between A and B compartments transiently increased in NPCs, and interactions within the B compartment increased during the NPC-to-neuron transition, supporting the notion that chromatin undergoes global compaction in association with differentiation (Figure 2B). Also consistent with this idea, the positive correlation between active histone marks [H3K4me1, H3K27ac (acetylated lysine-27 of histone H3), and H3K36me3] and the A compartment became weaker, whereas that between the inactive mark H3K9me3 and the B compartment became stronger, during neural (ESC-NPC-neuron) differentiation (Bonev et al., 2017). Regarding the inactive (B) compartment, as extreme cases, rod photoreceptor cells manifest heterochromatin aggregation in the center of the nucleus (Solovei et al., 2009), and postmitotic olfactory sensory neurons show pronounced compaction of olfactory receptor gene loci (Clowney et al., 2012; Le Gros et al., 2016). However, Hi-C results suggest that the compaction of heterochromatin domains may be a general feature of differentiating neurons and contribute to the stable silencing of unnecessary genes for differentiated neurons (Solovei et al., 2009; Clowney et al., 2012; Bonev et al., 2017). Given the changes in LADs during neural development (Peric-Hupkes et al., 2010), the downregulation of a lamin B receptor apparent during neuronal differentiation provides a possible common mechanism for this heterochromatin reorganization (Clowney et al., 2012; Solovei et al., 2013).

How then are regions in the A compartment regulated? High-level interactions within the A compartment in ESCs can be explained in part by long-range (>30 Mb) associations between active promoters, enhancers, and actively transcribed genes both *in cis* and *in trans* (Li et al., 2012; Schoenfelder et al., 2015; Tang et al., 2015; Bonev et al., 2017). In addition to 3C-based methods, a technique known as genome architecture mapping (GAM) can determine the proximity of genomic loci without cross-linking by ultrathin cryosectioning of nuclei followed by laser microdissection and DNA sequencing (Beagrie et al., 2017). GAM confirmed an abundance of long-range interactions, especially between “super-enhancers” [which are marked by extremely high levels of H3K27ac (Hnisz et al., 2013; Parker et al., 2013)] in ESCs. Super-enhancers are cell type specific and play key roles in cell fate determination (Hnisz et al., 2013). Given that they are enriched in binding elements for cell type-specific transcription factors (Hnisz et al., 2013), it is possible that homotypic interactions between these factors can induce the aggregation (high-density interaction) of super-enhancers. Moreover, whereas high-density contacts between active promoters were found to be independent of CTCF (Bonev et al., 2017), degradation of the cohesin component Rad21 resulted in an increase in the number of long-range interactions between super-enhancers (Rao et al., 2017), suggesting that the cohesin complex insulates long-range interactions between super-enhancers and thereby ensures the fidelity of cell type-specific gene expression patterns.

On the basis of classical immunocytochemical analyses, nuclear bodies, which are subcompartments within the nucleus, were hypothesized to serve as hubs for active or inactive gene loci (Rino et al., 2007; Sutherland and Bickmore, 2009; Padeken and Heun, 2014), although there was no genome-wide evidence to support this notion. A ligation-independent method known as split-pool recognition of interactions by tag extension (SPRITE) that relies on uniquely tagged cross-linked chromatin fragments to determine the proximity of genomic loci was recently introduced (Quinodoz et al., 2018). This method detects proximity between both DNA and RNA molecules and revealed that regions in the active (A) compartment preferentially interact with U1 spliceosomal RNA and Malat1 long noncoding RNA localized at nuclear speckles, whereas those in the inactive (B) compartment interact with rRNA localized at the nucleolus (Figure 2C). Consistent with these observations, the contact enrichment between gene bodies positively correlates with transcriptional level as well as with the numbers of exons and splicing events (Bonev et al., 2017). Given the contribution of nuclear bodies to neural development (Bernard et al., 2010; Hetman and Pietrzak, 2012), these results suggest that the dynamic rearrangements of A/B compartments during neural development may be dependent on or connected to changes in nuclear bodies.

### Global Changes in Polycomb Domains

In general, active and inactive histone modifications are associated with A and B compartments, respectively (Lieberman-Aiden et al., 2009; Rao et al., 2014). Interestingly, although H3K27me3, a modification deposited by Polycomb repressor complex 2 (PRC2), is generally considered an inactive histone mark, it is highly associated with the A compartment in ESCs and becomes associated more with the B compartment in neurons (Bonev et al., 2017; Figure 2D). This finding can be explained in part by the role of Polycomb group (PcG) proteins in the maintenance of developmental genes in the “poised” state in stem cells for later activation in response to differentiation-inducing cues (Azuara et al., 2006; Bernstein et al., 2006; Zhao et al., 2007). Such poised promoters tend to be “bivalent” in that they possess both active (H3K4me3) and inactive (H3K27me3) marks, and are thus included in the A compartment. Consistent with the notion that PcG proteins, including Ring1B – a major component of Polycomb repressor complex 1 (PRC1) – are associated with many poised developmental genes included in the A compartment in pluripotent stem cells and that such association is attenuated after differentiation, the genomic loci bound by Ring1B manifest strong interactions in ESCs but these interactions become progressively reduced during neural differentiation (Bonev et al., 2017). Furthermore, PcG protein-mediated chromatin interactions can take place beyond TAD boundaries and establish inter-TAD and inter-chromosomal associations in addition to those within TAD boundaries (Denholtz et al., 2013; Schoenfelder et al., 2015; Kundu et al., 2017). The global loss of PRC1-mediated, but H3K27me3-independent, long-range chromatin interactions during neural differentiation may therefore account in part for the global changes in chromatin architecture associated with this process. Conversely, a specific subset of Ring1B-mediated interactions becomes stronger during differentiation so as to allow for persistent repression of certain developmental genes associated with fate restriction (Bonev et al., 2017; Tsuboi et al., 2018). These inactive genes that are persistently silenced by PcG proteins are included in the B compartment. Mechanistically, PcG proteins can mediate high-density chromatin interactions *via* self-aggregation within and between PRC1 and PRC2 (Kim et al., 2002; Francis et al., 2004; Margueron et al., 2008; Eskeland et al., 2010; Grau et al., 2011; Isono et al., 2013). In particular, Phc protein components of PRC1 form nuclear nanoclusters in a manner dependent on polymerization activity of the SAM (sterile alpha motif) domain, with the formation of these clusters facilitating long-range chromatin interactions and persistent silencing (Isono et al., 2013; Wani et al., 2016; Tsuboi et al., 2018).

## Local (Intra- or Sub-TAD) Changes in 3D Genome Organization During Neural Differentiation

### Interactions Between Binding Sites of Neural-Specific Transcription Factors in NPCs and Neurons

**FIGURE 3:**
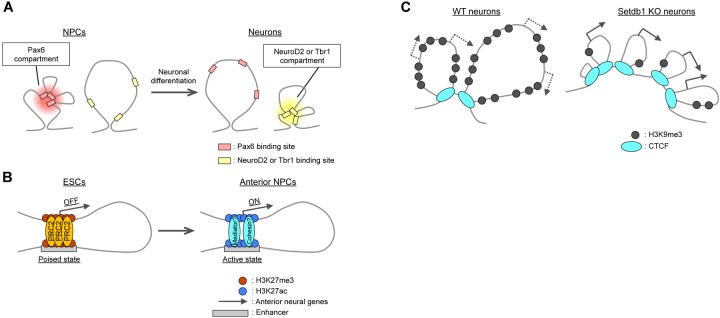
Local changes in 3D genome organization during neural differentiation. **(A)** Intra-TAD interactions between binding regions for cell type-specific transcription factors such as Pax6, NeuroD2, and Tbr1. **(B)** Polycomb (PRC2)-mediated interactions between promoters and poised enhancers lead to the activation of anterior neural genes during differentiation. **(C)** In cortical neurons, H3K9me3 deposition catalyzed by Setdb1 prevents aberrant CTCF binding at Pcdh gene clusters. Knockout (KO) of Setdb1 induces excessive insulation and upregulation of Pcdh gene expression.

Topologically associated domains constitute units of gene regulation (Alexander and Lomvardas, 2014; Dixon et al., 2015; Lupiáñez et al., 2015; Narendra et al., 2016; Symmons et al., 2016; Zhan et al., 2017), with most enhancer–promoter interactions taking place within TADs. High-resolution Hi-C or promoter-capture Hi-C analyses have confirmed that such interactions are highly cell type specific (Rao et al., 2014; Javierre et al., 2016; Bonev et al., 2017; Freire-Pritchett et al., 2017). For example, neuronal enhancers interact with their promoters more strongly in neurons than in ESCs and NPCs (Mifsud et al., 2015; Bonev et al., 2017). Chromatin immunoprecipitation (ChIP)-seq analyses have revealed a link between intra-TAD interactions and cell type-specific transcription factors such as the NPC-specific Pax6 and the immature neuron- and mature neuron-specific NeuroD2 and Tbr1, respectively (Figure 3A). The interactions of Pax6-bound sites were thus stronger in NPCs than in neurons or ESCs, whereas those of NeuroD2- or Tbr1-bound sites were stronger in neurons than in NPCs or ESCs (Bonev et al., 2017). Transcription factors may also organize the co-regulation of target genes through homotypic interactions or association with partner molecules such as the BAF chromatin remodeling complex for Pax6 (Ninkovic et al., 2013; Manuel et al., 2015).

### PcG Protein-Mediated Enhancer–Promoter Interactions at Neural Gene Loci in ESCs

Polycomb group proteins generally mediate repression of gene expression, as mentioned above. However, recent studies have revealed that these proteins may contribute to gene activation *via* the establishment of enhancer–promoter interactions. PRC1 (Ring1) can mediate the association of a midbrain-specific enhancer and the promoter of the *Meis2* gene during midbrain development, with the subsequent dissociation of PcG proteins resulting in the activation of *Meis2* expression in the midbrain (Kondo et al., 2014; Yakushiji-Kaminatsui et al., 2016). PcG proteins were found to play a similar role in the establishment of “poised” enhancers in ESCs. The poised enhancers were defined by the presence of the histone acetyltransferase p300 and H3K27me3 and the absence of H3K27ac and H3K4me3, and neural genes, especially anterior neural genes, were found to be enriched in poised enhancers in ESCs (Cruz-Molina et al., 2017). Importantly, poised enhancers physically contact their target genes in a PRC2-dependent manner, and the PRC2 components Suz12 and Eed are necessary for the induction of anterior neural genes in NPCs (Figure 3B). These findings point to the essential role of PcG proteins in the generation of permissive chromatin topology at such gene loci before their activation, although the molecular basis of their differential roles in gene activation and suppression remains to be clarified.

The preferential regulation of anterior neural genes by poised enhancers in ESCs (Cruz-Molina et al., 2017) *per se* is an intriguing finding. Classical developmental models propose that epiblast cells *in vivo* and ESCs *in vitro* are fated toward the neural lineage by “default” (that is, in the “absence” of extrinsic signals) (Levine and Brivanlou, 2007; Gaspard and Vanderhaeghen, 2010). Moreover, induced neural progenitors initially manifest anterior characteristics (that is, those of the forebrain), which must be overridden by extrinsic cues for the induction of more posterior neural fates (such as those of the spinal cord). The readiness of anterior neural genes to be expressed due to their association with poised enhancers in ESCs may explain in part the propensity for default differentiation to an anterior neural lineage.

### TAD Boundary Formation in Neural Cells

As mentioned above, most TAD boundaries are conserved between ESCs and neural cells, but a fraction of TAD boundaries appears to emerge and disappear during neural differentiation (Bonev and Cavalli, 2016) – although the interpretation of TAD boundaries depends on the precise definition of TADs (Dixon et al., 2016). Of note, these developmentally regulated TAD boundaries correlate with H3K4me1-positive enhancers (Dixon et al., 2015) and active gene marks (Bonev et al., 2017) as well as with the presence of cohesin, but not that of CTCF (Bonev et al., 2017). Indeed, the emergence of new boundaries in NPCs was found to be associated with Zfp608- and Sox4-dependent transcription, although forced induction of such transcription with the use of the dCas9 system was not sufficient to induce a new TAD boundary (Bonev et al., 2017).

### Relevance of TAD Boundaries to Regulation of Pcdh Gene Clusters

Topologically associated domain boundaries can play a role in the regulation of neural genes, most notably in Protocadherin (Pcdh) gene clusters. Pcdh proteins regulate axonal targeting, synapse formation, and dendritic arborization through their homophilic trans-interactions (Yagi, 2012; Chen and Maniatis, 2013). The vast diversity of neurons is generated in part by the stochastic and combinatorial expression of the clustered Pcdh genes, which include Pcdhα, Pcdhβ, and Pcdhγ clusters aligned *in cis*. *In situ* Hi-C experiments with NeuN-positive mouse neocortical neurons revealed that the Pcdh gene clusters are organized as multiple small TADs (∼100 kb in length) nested into a larger TAD that encompasses at least 1.2 Mb. The 5′ end of the Pcdhα cluster is bound to the 3′ end of the Pcdhγ cluster (Jiang et al., 2017). This TAD structure appears to be important for proper regulation of Pcdh genes, given that knockout of CTCF disrupted TADs at this locus and resulted in the aberrant expression of Pcdh genes (Hirayama et al., 2012; Sams et al., 2016). The unique TAD structure of Pcdh gene clusters was also apparent in neurons derived from human induced pluripotent stem cells (iPSCs). Interestingly, a risk haplotype for schizophrenia (according to the Psychiatric Genomic Consortium) has been found to be genetically linked to the 5′ end of the human Pcdhα gene cluster (Schizophrenia Working Group of the Psychiatric Genomics Consortium, 2014). Forced dCas9-mediated localization of KRAB or VP64 transcriptional repressor or activator domains, respectively, at the risk gene locus in human iPSC-derived NPCs resulted in dysregulation of Pcdh gene transcription (Jiang et al., 2017). Given the neurodevelopmental functions of Pcdh proteins, an aberrant TAD structure of the Pcdh gene clusters could potentially contribute to the development of schizophrenia.

With regard to the mechanism responsible for the TAD structure of Pcdh gene clusters, deposition of H3K9me3 by the histone methyltransferase Setdb1 (also known as Kmt1e or ESET) (Schultz et al., 2002) appears to play an essential role. Ablation of Setdb1 in neocortical neurons reduced the level of H3K9me3 and increased the binding of CTCF at the Pcdh gene clusters, resulting in the formation of only small TADs without the large-scale interaction normally apparent between the borders of the clusters (Figure 3C; Jiang et al., 2017). Cytosine methylation (5mC) was shown to inhibit the binding of CTCF (Renda et al., 2007; Wang et al., 2012), although this finding is still under debate (see Bonev and Cavalli, 2016). Setdb1 ablation reduced 5mC levels at several residues in the Pcdh gene clusters, which thus may account for the increased CTCF binding and aberrant insulation within these clusters.

Regulation of CTCF binding and TAD structure by Setdb1 is not restricted to Pcdh gene clusters. Loss of Setdb1 in neocortical neurons thus resulted in the emergence of more than 3000 ectopic CTCF-binding sites (Jiang et al., 2017). Setdb1 has also been shown to contribute to the development of several tissues including the mouse neocortex (Tan et al., 2012; Liu et al., 2014; Eymery et al., 2016; Kim et al., 2016; Takikita et al., 2016). Ablation of Setdb1 altered the differentiation potential of neocortical NPCs by reducing neurogenic potential and increasing astrogenic potential. Of interest, transcriptome analysis of Setdb1-deficient NPCs revealed ectopic expression of genes of nonneural lineages as well as of transposons (Tan et al., 2012), implicating Setdb1 in repression of these genes, possibly mediated by inhibition of unwanted CTCF binding and consequent promotion of proper formation of TAD structures in addition to its role in heterochromatin formation through H3K9me3. CTCF binding is also regulated by other factors including YY1, which may control enhancer–promoter interactions and transcription in NPCs (Beagan et al., 2017; Weintraub et al., 2017), although the ubiquitously expressed YY1 alone may not be able to account for cell type-specific CTCF regulation.

## Conclusion and Future Directions

The regulation of 3D chromatin structure has been studied with regard to its role in determination of gene transcription patterns. New technologies such as high-resolution 3C-based methods have revealed that neural development is accompanied by changes in genome organization at the levels of both interactions between large compartments and local interactions such as those between enhancers and promoters. Such advances in basic knowledge concerning chromatin structure will facilitate our understanding of the mechanisms and relevance of chromatin regulation during neural development and the pathogenesis of related diseases. Given the heterogeneity of NPCs and neurons, analyses at the single-cell level will be especially important for studies of neural development, and the recent implementation of advanced single-cell RNA-seq, ChIP-seq, DamID, ATAC-seq, and Hi-C technologies should prove highly informative in this regard (Nagano et al., 2013; Shalek et al., 2014; Buenrostro et al., 2015; Kind et al., 2015; Macosko et al., 2015; Rotem et al., 2015; Corces et al., 2016; Stevens et al., 2017). The spatial and functional nature of the relation between chromatin domains and nuclear bodies, the nuclear lamina, and other aspects of nuclear architecture also await clarification in future studies (Sutherland and Bickmore, 2009; Quinodoz et al., 2018). Recent developments in advanced microscopic technology, including super-resolution and electron microscopies, as well as in live-cell imaging of specific genomic loci with the use of zinc-finger nuclease, transcription activator-like effector nuclease (TALEN), or CRISPR (clustered regularly interspersed short palindromic repeats)–Cas9 systems may uncover novel principles of 3D organization and genomic localization in the nucleus (Chen et al., 2016; Ricci et al., 2017). We focused in this review on the early developmental process of neural differentiation, but it will also be of interest to determine how chromatin architecture is regulated during neuronal maturation and in association with neural plasticity triggered by changes in neuronal activity (Wittmann et al., 2009; Frank et al., 2015; Thakurela et al., 2015; de la Torre-Ubieta et al., 2018; Gallegos et al., 2018; Preissl et al., 2018).

As suggested in the case of the Pcdh gene clusters, aberrant changes in 3D chromatin structure may give rise to neurodevelopmental disorders (Mitchell et al., 2014). Indeed, mutations in the genes for cohesin components are known to be responsible for Cornelia de Lange syndrome in humans, which is associated with mental retardation (Krantz et al., 2004; Tonkin et al., 2004; Deardorff et al., 2007, 2012; Fujita et al., 2017). Mutations in CTCF and Setdb1 genes also cause severe neural developmental defects in mice (Watson et al., 2014; Sams et al., 2016). Although access to human tissue is limited, the organization of the human genome in both the developing and adult human brain has recently been investigated by Hi-C analyses (Won et al., 2016). Such studies as well as those of neurons derived from iPSCs of patients with neurodevelopmental disorders should provide insight into the pathogenesis of these conditions as well as a basis for the development of new therapeutic strategies.

## Author Contributions

YK and YG wrote the manuscript.

## Conflict of Interest Statement

The authors declare that the research was conducted in the absence of any commercial or financial relationships that could be construed as a potential conflict of interest.
